# Identification of Novel Immunogenic Proteins from *Mycoplasma bovis* and Establishment of an Indirect ELISA Based on Recombinant E1 Beta Subunit of the Pyruvate Dehydrogenase Complex

**DOI:** 10.1371/journal.pone.0088328

**Published:** 2014-02-10

**Authors:** Zhenhong Sun, Ping Fu, Kai Wei, Haiyan Zhang, Yuewei Zhang, Jian Xu, Fei Jiang, Xu Liu, Wei Xu, Wenxue Wu

**Affiliations:** Key Laboratory of Animal Epidemiology and Zoonosis, Ministry of Agriculture, College of Veterinary Medicine, China Agricultural University, Beijing, P. R. China; INIAV, I.P.- National Institute of Agriculture and Veterinary Research, Portugal

## Abstract

The pathogen *Mycoplasma bovis* (*M. bovis*) is a major cause of respiratory disease, mastitis, and arthritis in cattle. Screening the key immunogenic proteins and updating rapid diagnostic techniques are necessary to the prevention and control of *M. bovis* infection. In this study, 19 highly immunogenic proteins from *M. bovis* strain PD were identified using 2-dimensional gel electrophoresis, immunoblotting and MALDI-TOF/TOF MS. Of these 19 proteins, pyruvate dehydrogenase E1 component beta subunit (PDHB) showed excellent immune reactivity and repeatability. PDHB was found to be conserved in different *M. bovis* isolates, as indicated by Western blot analysis. On the basis of these results, a rPDHB-based indirect ELISA (iELISA) was established for the detection of serum antibodies using prokaryotically expressed recombinant PDHB protein as the coating antigen. The specificity analysis result showed that rPDHB-based iELISA did not react with other pathogens assessed in our study except *M. agalactiae* (which infects sheep and goats). Moreover, 358 serum samples from several disease-affected cattle feedlots were tested using this iELISA system and a commercial kit, which gave positive rates of 50.8% and 39.9%, respectively. The estimated Kappa agreement coefficient between the two methods was 0.783. Notably, 39 positive serum samples that had been missed by the commercial kit were all found to be positive by Western blot analysis. The detection rate of rPDHB-based iELISA was significantly higher than that of the commercial kit at a serum dilution ratio of 1∶5120 to 1∶10,240 (*P*<0.05). Taken together, these results provide important information regarding the novel immunogenic proteins of *M. bovis*. The established rPDHB-based iELISA may be suitable for use as a new method of antibody detection in *M. bovis*.

## Introduction

*Mycoplasma bovis* (*M. bovis*) is a major but often overlooked pathogen. It mainly causes respiratory disease, mastitis, arthritis, keratoconjunctivitis, and otitis. *M. bovis* was first isolated from a case of severe mastitis in cattle in 1961 [1]. It has since been reported to be connected with bovine respiratory disease [2]. In China, it was first isolated in 2008, from the lungs of calves infected with pneumonia [3]. This disease exists worldwide today. In Europe, about 25–33% of cases of calf pneumonia are caused by or associated with *M. bovis*. In the U.S., *M. bovis* is responsible for annual losses of USD 140 million resulting from bovine respiratory disease and breast disease, with a maximum infection rate of up to 70% per cattle feedlot [4]–[6].

Under natural conditions, *M. bovis* infection is difficult to identify and easy to confuse with contagious pleuropneumonia because their clinical symptoms and pathologic changes are very similar. This leaves laboratory differential diagnosis as the best available way to identify *M. bovis* infection. Generally, serological diagnosis is more sensitive than *M. bovis* isolation, especially for the chronic cases or animals treated with antibiotics [5]. Currently, a few commercial indirect ELISA kits have been used for this purpose. The commonly used are the *Mycoplasma bovis* Antibody Test Kit which is produced by Canada’s Biovet Company and Bio-X *Mycoplasma bovis* ELISA Kit produced by Belgium’s Bio-X Diagnostics Company. Most kits are based on whole-cell proteins, and the effects with respect to the detection of *M. bovis* infection in different geographic regions have yet to be verified. However, the use of specific, highly pure antigens with high affinity to antibodies as coating antigens may render the diagnosis more accurate.

Early reported *M. bovis* immunogenic proteins involved variable surface proteins (Vsps). These membrane-surface antigens can vary in phase and size. This involves high-frequency rearrangements of the DNA region encoding the Vsp genes. These rearrangements play a major role in evading the immune system of the host [7], [8]. In recent years, several relatively conserved immunogenic proteins have been discovered. These include the conserved P26 [9] and P48 [10] lipoproteins, heat-shock proteins [11], and glyceraldehyde-3-phosphate dehydrogenase (GAPDH) [12]. These proteins may be suitable for use as candidate antigens for diagnosis and subunit vaccines against *M. bovis*. Currently, only a few of the immunogenic proteins of *M. bovis* are well understood. More immunogenic proteins must be identified to facilitate development of more effective approaches to both the diagnosis and prevention of *M. bovis*.

Proteome analysis is a useful complementary method of studying pathogens. It facilitates genome annotation and protein identification [13], [14]. Immunoproteomics, which combines conventional proteomics with serology, is a powerful method of identifying immunodominant antigens that have diagnostic and protective value [15]. In this study, 19 immunogenic proteins were identified in a strain of *M. bovis* that had been isolated in China. These proteins were identified using immunoproteomics with four positive sera (Table S1) collected from the disease-affected cattle feedlots in different provinces. An iELISA method of detecting serum antibodies was established based on prokaryotically expressed antigen protein E1 beta subunit of the pyruvate dehydrogenase complex (PDHB). It was found to be highly sensitive and specific.

## Results

### Two-dimensional gel electrophoresis (2-DE) and immunoblotting

**Figure 1:**
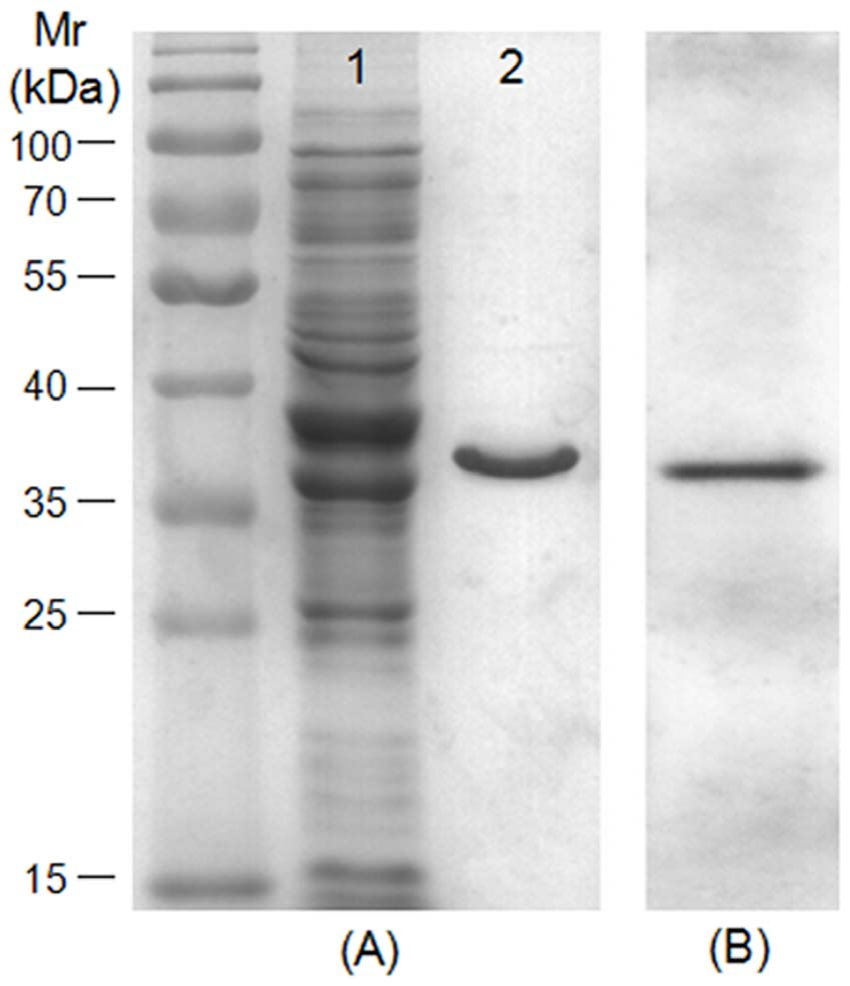
Extraction of the whole-cell proteins of *M. bovis* strain PD and expression of rPDHB protein. (A1) The extracted whole-cell proteins were separated by SDS-PAGE. (A2) Purity analysis of the recombinant His-tagged PDHB protein by SDS-PAGE. (B) Purified rPDHB protein was subjected to Western blot analysis using an anti-His-tag antibody.

**Figure 2:**
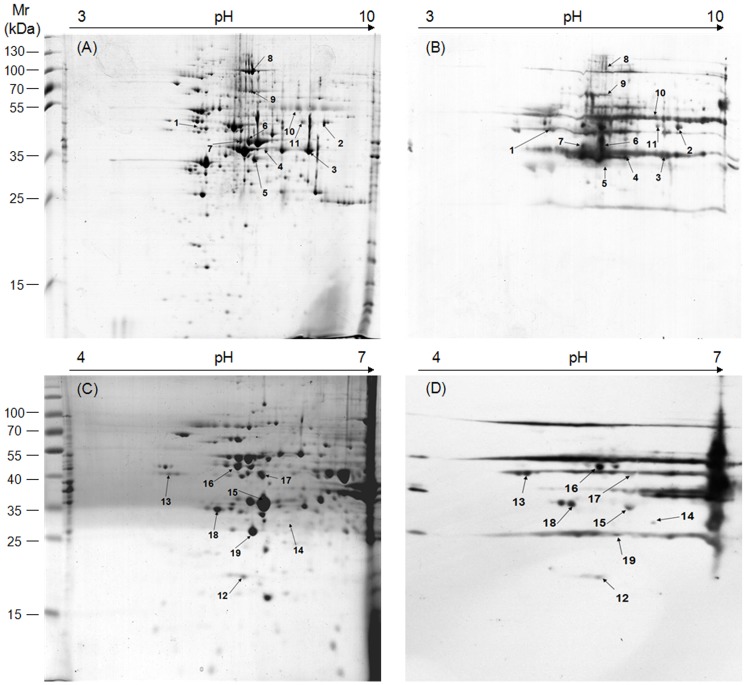
Two-dimensional gel electrophoresis (2-DE) and immunoblotting of the whole-cell proteins of *M. bovis* strain PD. First, 350 µg and 100 µg of protein were separated by IEF using (A) a pH 3–10 IPG strip and (C) a pH 4–7 IPG strip, respectively. This was followed by SDS-PAGE on 12% gels and staining with Coomassie brilliant blue R-350. Immunoblotting was performed using each of the four antisera (Table S1) from naturally infected cattle with three replicates. The immunoreactive protein spots on PVDF membranes B and D contained all spots with good reproducibility as identified by each of the four positive sera. These corresponded to the proteins separated by 2-D gels A and C, respectively. pI values are shown on top, and the standard molecular weights are shown to the left of the gels. The spot numbers correspond to those identified by MS and listed in Table 1.

To separate the whole-cell proteins of *M. bovis*, IPG strips of different pH ranges (pH 3–10 and pH 4–7) were used for 2-DE (Fig. 1A). This 2-DE process was shown to be reproducible by running different batches of protein samples to each IPG strip three times (data not shown). About 570 prominent protein spots, with molecular weights ranging from 15 to 130 kDa, were detected in Coomassie brilliant blue R-350 stained 2-DE gels with pH 3-10 IPG strips (17 cm, NL) (Fig. 2A). The isoelectric points (pI) of most proteins were concentrated within the range of 4–7. Taking into account that there are 765 proteins encoded by the genome of *M. bovis* reference strain PG45, the rate of coverage of proteins separated in the present study was found to be 74.5%. According to the isoelectric points of most proteins, IPG strips of pH 4–7 were selected for use in the following tests to facilitate better separation of the proteins (Fig. 2C).

The proteins separated in 2-DE gels with pH 3–10 and 4–7 IPG strips were blotted onto PVDF membranes, respectively. Then, each immunoblot assay was performed using the strongly positive sera A, B, C, and D (Table S1), collected from naturally infected cattle. About 60 and 35 spots were found to be positive on the two PVDF membranes, as shown in Figs. 2B and 2D. These immunoreactive protein spots showing good reproducibility were identified using each strongly positive serum. No positive spot was detected in the test using the negative sera (data not shown).

### Immunogenic proteins identified using MALDI-TOF/TOF MS and bioinformatics analysis

At first, 21 and 11 immunoreactive protein spots with good reproducibility were excised from 2-DE gels stained with Coomassie with pH 3–10 and 4–7 IPG strips, respectively. MALDI-TOF/TOF MS analysis indicated the presence of 31 protein spots, corresponding to a total of 19 different proteins. Of these, 12 proteins corresponded to single spots and 7 proteins were represented by multiple immunoreactive spots, indicating post-translational modifications such as chemical modification and proteolytic cleavage. The information regarding the identity, isoelectric point, molecular weight, identification score, and protein score of these immunogenic proteins are shown in Table 1.

**Table 1:** *M. bovis* proteins identified by mass spectrometry and reactions with sera from naturally infected cattle in immunoblotting experiments.

Spot no.	Protein	NCBI ID	pI	MW (kDa)	Identificationscores	Protein score C.I. %^a^	COG^b^
1	Peptide chain release factor 1	gi|313678884	5.30	40.43	842	100	J
2	Pyrimidine-nucleoside phosphorylase	gi|313678400	8.09	47.26	1020	100	F
3	Glyceraldehyde-3-phosphate dehydrogenase, type 1	gi|313678191	7.70	36.85	814	100	G
4	Pyruvate dehydrogenase E1 component subunit alpha	gi|313678230	6.13	41.33	435	100	C
5	L-lactate dehydrogenase	gi|313678425	6.35	35.02	962	100	C
6	Oxidoreductase, zinc-binding dehydrogenase family	gi|313678628	6.51	37.96	778	100	E
7	Acetate kinase	gi|339320670	6.16	44.27	889	100	C
8	D-xylulose 5-phosphate/D-fructose 6-phosphate phosphoketolase	gi|313678257	6.30	89.91	929	100	G
9	Pyruvate dehydrogenase complex, E3 component, dihydrolipoamide dehydrogenase	gi|313678233	6.50	58.80	569	100	C
10	Adenylate kinase	gi|344204920	8.53	24.33	76	99.91	F
11	Thymidine phosphorylase(TDRPASE)	gi|339321078	7.60	47.28	995	100	F
12	Translation elongation factor P	gi|313678493	5.36	21.00	148	100	J
13	Cell division protein ftsZ	gi|339320924	4.86	41.95	110	100	D
14	Thioredoxin-disulfide reductase	gi|313678228	5.50	33.89	164	100	O
15	Pyruvate dehydrogenase E1 component subunit beta	gi|313678231	5.44	36.18	1070	100	C
16	Phosphopentomutase	gi|313678465	5.32	44.02	854	100	G
17	Lipoyltransferase and lipoate-protein ligase	gi|313678142	5.43	39.78	693	100	H
18	Translation elongation factor Ts	gi|313678643	5.23	32.67	438	100	J
19	Purine nucleoside phosphorylase	gi|313678401	5.33	25.92	956	100	F

Protein spots from 2-DE were sequenced using MALDI-TOF/TOF MS and identified by searching mycoplasma databases using the MASCOT search engine 2.2. A GPS explorer protein confidence index ≥95% were used for further manual validation.

a C.I. %: the confidence interval for the protein score.

b COG database functional classes: (C) energy production and conversion, (D) cell cycle control, cell division, chromosome partitioning, (E) amino acid transport and metabolism, (F) nucleotide transport and metabolism, (G) carbohydrate transport and metabolism, (H) coenzyme transport and metabolism, (J) translation, ribosomal structure and biogenesis, (O) post-translational modification, protein turnover, chaperones.

PSORTb analysis predicted that 18 of the 19 immunogenic proteins would be located in the cytoplasm, and the cell division protein ftsZ might be located at multiple sites, including the cytoplasm and cytoplasmic membrane. Most of the proteins identified in this way are enzymes involved in cell metabolism, cell structure and host cell invasion. According to the clusters of orthologous groups (COG) functional classification system, these proteins were assigned to the metabolism, cellular processing and signaling, and information storage and processing groups. Specifically, these proteins were found to be involved in energy production and conversion (spots 4, 5, 7, 9, and 15); carbohydrate transport and metabolism (spots 3, 8, and 16); amino acid transport and metabolism (spot 6); nucleotide transport and metabolism (spots 2, 10, 11, and 19); coenzyme transport and metabolism (spot 17); cell cycle control, cell division, and chromosome partitioning (spot 13); posttranslational modification, protein turnover, and chaperoning (spot 14), and translation, ribosomal structure and biogenesis (spots 1, 12, and 18). Pyruvate dehydrogenase E1 component alpha subunit (PDHA, spot 4), pyruvate dehydrogenase E1 component beta subunit (PDHB, spot 15), and GAPDH (spot 3) have also been reported to participate in the process of adhesion to host cells.

PDHB is usually considered a cytoplasmic protein, but it has been detected on the surfaces of some bacteria, and the antigenicity of PDHB in other mycoplasma species has also been demonstrated [16]–[18]. In the present study, the immunogenicity of PDHB was proved to be reproducible by Western blot analysis using strongly positive sera. Specific primers have been designed to amplify PDHB genes from different *M. bovis* isolates. The amino acid sequence of PDHB of the *M. bovis* strain PD showed over 99.7% homology with PDHBs from other *M. bovis* isolates, about 98.2% homology with the PDHB from *M. agalactiae* (which infects sheep and goats) and 66.2% homology with those of other mycoplasma species (isolated from cattle, sheep and goats) (Table 2). These data showed the protein PDHB to be structurally conserved within the *M. bovis* cluster.

**Table 2:** Homology of PDHB from the *M. bovis* strain PD and other mycoplasmas.

Mycoplasmas	NCBI Reference sequence	Homology
*Mycoplasma bovis* PG45	YP_004055971.1	100.0%
*Mycoplasma bovis* Hubei-1	YP_004683146.1	99.7%
*Mycoplasma bovis* HB0801	YP_006470739.1	99.7%
*Mycoplasma agalactiae* PG2	YP_001256238.1	98.2%
*Mycoplasma bovigenitalium*	WP_004420033.1	66.2%
*Mycoplasma ovipneumoniae*	WP_010321080.1	58.5%
*Mycoplasma mycoides subsp. capri* str. GM12	ACU79371.1	47.9%
*Mycoplasma mycoides subsp. mycoides* SC str. PG1	NP_975265.1	47.9%
*Mycoplasma capricolum subsp. capricolum* ATCC 27343	YP_424213.1	47.3%

Mycoplasmas shown on the list were selected using the NCBI BLAST server, basing on the principle of recent homology. Then the amino acid sequences of these PDHBs were downloaded from NCBI, and DNAstar software (version 5.0) was used to analyze the homology.

### Expression of *M. bovis* PDHB

The 987 bp gene encoding PDHB was amplified from the genome of *M. bovis* strain PD and cloned into a prokaryotic vector. Then recombinant PDHB (rPDHB) containing six histidine residues was expressed in *E. coli*, as illustrated in Fig. 1A. The Western blot analysis result indicated the presence of an immunoreactive band of about 37 kDa corresponding to the rPDHB (Fig. 1B), with a slightly higher molecular mass than the native protein of *M. bovis*.

### Antigenicity analysis of *M. bovis* PDHB

**Figure 3:**
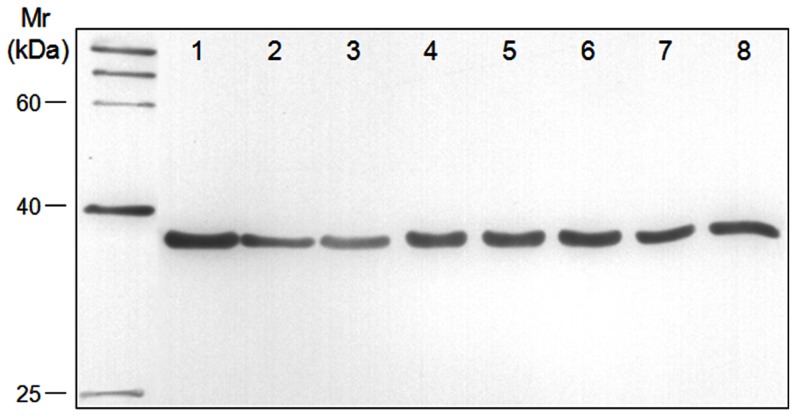
Antigenicity of PDHBs from different *M. bovis* isolates. The whole-cell proteins of eight *M. bovis* isolates were separated by SDS-PAGE, blotted onto a PVDF membrane, and subjected to Western blot analysis with a rabbit anti-rPDHB polyclonal antibody. The positions of molecular mass markers are indicated at the left in kDa. Lanes 1–8: *M. bovis* strain PD, PG45, SD-2, Hubei-1, HRB-1, GY-7, GY-14, and WF-3, respectively.

In order to examine whether the antigenicity of *M. bovis* PDHB was species-specific, Western blot analysis was performed to assess the reactivity of the prepared rabbit anti-rPDHB polyclonal antibody and the whole-cell proteins of eight *M. bovis* isolates. As shown in Fig. 3, all eight *M. bovis* strains isolated from different regions reacted with the rabbit anti-rPDHB polyclonal antibody. For the reaction with *M. bovirhinis*, *M. ovipneumoniae*, *M. agalactiae*, bovine viral diarrhea virus (BVDV), bovine parainfluenza virus type 3 (BPIV3), and infectious bovine rhinotracheitis virus (IBRV), all these pathogens failed to be recognized by the anti-rPDHB polyclonal antibody except *M. agalactiae* (Fig. S1). All pathogens in the present study were identified using specific PCR, as shown in Fig. S2. The results indicated that the antigenicity of *M. bovis* PDHB was similar to that of *M. agalactiae* PDHB but markedly different from that of other pathogens.

### Establishment of rPDHB-based iELISA

According to the immunogenicity and antigenicity of *M. bovis* PDHB, a rPDHB-based iELISA was established to confirm the feasibility of rPDHB as a diagnostic antigen. Standard positive and negative sera were used to optimize the reaction conditions. Eventually, the best reaction conditions were selected as follows: the concentration of rPDHB used for coating was 100 ng/well, blocking buffer was 10% sheep serum, the best dilutions of serum sample and secondary antibody were 1∶160 and 1∶2000 (v/v), respectively, and the cutoff value was 0.316.

To examine the specificity of this iELISA, the prepared positive sera of other pathogens, including *Mycoplasma mycoides subsp. mycoides* SC (*Mmm*SC), *M. agalactiae*, *M. bovirhinis*, *M. ovipneumoniae*, BVDV, BPIV3, and IBRV, were tested. According to the detection results of all the positive sera listed above, only the sera against *M. agalactiae* reacted with *M. bovis* rPDHB (mean OD was 0.578).

### Performance of rPDHB-based iELISA and a commercial kit

rPDHB-based iELISA and the commercial kit were both used to test 358 serum samples collected from cattle feedlots in different provinces. As shown in Table 3, 182 and 143 sera were confirmed to be positive for anti-*M. bovis* antibodies by iELISA and the commercial kit. The estimated Kappa agreement coefficient between the two detection methods was 0.783. Then Western blot analysis was performed to confirm the incompatible sera by both methods using the whole-cell proteins of *M. bovis* strain PG45. Notably, 39 positive serum samples that had been missed by the commercial kit were all correctly found to be positive by Western blot analysis. All negative samples detected by rPDHB-based iELISA were also found to be negative by the commercial kit. The results suggested that the detection rate of rPDHB-based iELISA was higher than that of the commercial kit currently used for *M. bovis* antibody detection.

**Table 3:** Detection rates of rPDHB-based iELISA and the commercial kit.

No. of sera	Detection results		
	Commercial kit^a^	rPDHB-based iELISA	Western blot^b^
19	4+	Positive	NT^c^
71	3+	Positive	NT
38	2+	Positive	NT
15	1+	Positive	NT
176	Negative	Negative	NT
39	Negative	Positive	Positive
Detection rate (%)^d^	39.9 (143/358)	50.8 (182/358)	
Kappa	0.783		

a Positive results were classified from 1+ to 4+ according to the kit protocol.

b Western blot analysis was performed to confirm that the sera were incompatible. It was performed using the whole-cell proteins of *M. bovis* strain PG45.

c NT, not tested.

d Detection rate  =  (number of positive samples/total number of samples)×100.

To compare the sensitivity of two methods, 140 positive and 20 negative sera were selected, diluted, and detected with the commercial kit and rPDHB-based iELISA. As shown in Table 4, the highest dilution ratios of iELISA and commercial kit for detecting all 140 positive serum samples were both 1:640. However, iELISA was able to detect 127/140 positive sera at dilution ratio of 1:2560, and the detection rate of commercial kit was only 51/140. The difference was statistically significant (*P*<0.05). Moreover, the detection rate of rPDHB-based iELISA was significantly higher than that of the commercial kit at a serum dilution ratio of 1∶5120 to 1∶10,240 (*P*<0.05). In addition, iELISA was still competent to detect positive sera at dilution ratio as high as 1∶10,240 and showed a detection rate of 12/140, but the highest dilution ratio for the commercial kit was 1∶5120. The data suggested that the sensitivity of rPDHB-based iELISA was higher than that of the commercial kit.

**Table 4:** Sensitivity of rPDHB-based iELISA and the commercial kit.

Detection methods	Dilutions of the sera						
	1:320	1:640	1:1280	1:2560	1:5120	1:10240	1:20480
Commercial kit(no. of positive/total no.)	140/140^a^	140/140^a^	124/140^b^	51/140^c^	7/140^d^	0/140^e^	0/140^e^
rPDHB-based iELISA(no. of positive/total no.)	140/140^a^	140/140^a^	131/140^b^	127/140^b^	49/140^c^	12/140^d^	0/140^e^

Different lowercase letter superscripts indicate significant differences (*P*<0.05).

## Discussion

*M. bovis* has caused severe losses to the worldwide cattle industry. Currently, only a few *M. bovis* vaccines, including bacterin and autogenous vaccines, have been approved in the United States. However, most bacterin-based vaccines cannot provide complete protection. In some cases, vaccination did not bring down the morbidity and mortality but even aggravated the symptoms instead [19]–[21]. For this reason, laboratory diagnosis is of great significance to the prevention and treatment of *M. bovis* infection. Because *M. bovis* infection is often latent and the bacterium is seldom shed from healthy cattle, serological detection of *M. bovis* antibody, which can last for several months and can be detected at high levels by ELISA, is considered a more reliable method of diagnosis of *M. bovis* infection [5]. However, commercial kits based on the whole-cell proteins currently used for serological diagnosis of *M. bovis* infection cannot ensure the detection of the variegated *M. bovis* isolates, and the development of ELISA using the specific conserved antigen with higher specificity and sensitivity is considered a promising alternative. Therefore, the exploitation of *M. bovis* proteins with excellent immunogenicity is necessary to the renewal and improvement of *M. bovis* diagnostic techniques. In the present study, immunoproteomics was used to screen the immunogenic proteins from a *M. bovis* strain isolated in China. Information regarding the immunogenic proteins identified in this way can provide a reference for the diagnosis of *M. bovis* infection.

Immunoproteomics involves combining 2-DE, immunoblotting, and mass spectrometry for analysis of functional proteins. In recent years, the immunoproteomics approach has seen increasingly widespread use in the diagnosis and vaccine research of important livestock pathogenic mycoplasmas, such as *Mycoplasma capricolum subsp. capripneumoniae* (Mccp), *M. hyopneumoniae*, and *Mmm*SC [15], [18], [22]. However, it has seen less use in research into *M. bovis*. Thomas et al. performed 2-DE and MS to analyze the protein expression differences between the 7th and 116th generation of *M. bovis* strains [23]. Then an undiscovered adhesive protein as a new member of the Vsps family was identified. The study also showed that the phenotype of *M. bovis* may change after a long-term culture *in vitro*. For this reason, only the first three generations of *M. bovis* field-isolate strain PD were used for the preparation of protein samples in the present study. It was to prevent any change in *M. bovis* antigen proteins caused by long-term culture *in vitro* from affecting the results. Moreover, whole-cell proteins served as the protein sample for 2-DE instead of membrane proteins to prevent the loss of important cytoplasmic antigen proteins [12]. Joerg et al. demonstrated the importance of antiserum selection in the immunogenic protein screening process [15]. They screened out 24 immunogenic proteins using serum obtained from the cattle that had been experimentally infected with *Mmm*SC B237, but 13 additional proteins were detected when serum from the cattle with clinical acute onset was used. It indicated that the antibody induced in the naturally infected cattle is indispensable to the screening of immunogenic proteins of *M. bovis*. For this reason, strongly positive antisera from the cattle with natural onset were used in the present study for Western blot analysis to ensure that as many immunogenic proteins as possible would be recognized.

In the present 2-DE assay, about 570 and 338 prominent protein spots were separated in the gels with pH 3–10 and 4–7 IPG strips, respectively. According to the information provided by NCBI, there are 765 proteins encoded by *M. bovis* genome, which means that the present study acquired a proteome coverage of over 70%. It is similar to that observed in proteomic studies of other mycoplasma species [14], [15], [24]. Later, 19 immunogenic proteins were identified by Western blot and MALDI-TOF/TOF MS analysis. Most of these proteins were cytoplasmic proteins that were mainly involved in cell metabolism, cellular processes and signaling, and information storage and processing. Some of them were also related to adherence and invasion of host cells. Remarkably, some similar immunogenic proteins from other mycoplasma species have already been reported. These included PDHA, PDHB, GAPDH, L-lactate dehydrogenase (LDH), and translation elongation factors [15], [22], [25]. GAPDH is a highly conserved protein, and one of the features of GAPDH is its presence on the cell surface of several prokaryotic and eukaryotic cells, where it is able to bind mucin perhaps contributing to adherence to the epithelia [26]. In 2007, a study performed by Perez-Casal and Prysliak suggested that *M. bovis* GAPDH is an important immunogenic protein and might be a good candidate for diagnosis and vaccines [27]. However, they subsequently found that although cattle vaccinated with a subunit vaccine based on GAPDH produced high titers of IgG1 antibodies, there were few differences in the number of lung lesions and survival rate after challenge with a combination of three *M. bovis* isolates [12]. It indicates that screening and exploiting new immunogenic proteins are very necessary to the diagnosis and prevention of *M. bovis* infection. EF-Tu, EF-Ts, and EF-G are three factors usually involved in the protein translation process in prokaryotic cells. Elongation factor thermo stable proteins (EF-Ts) serve as guanine nucleotide exchange factors for elongation factor thermo unstable proteins (EF-Tu), catalyzing the release of guanosine diphosphate from EF-Tu. It allows EF-Tu to bind to a new guanosine triphosphate molecules, release EF-Ts, and go on to catalyze another aminoacyl tRNA addition. The EF-Tu proteins of many pathogens, including mycoplasmas, bacteria, and parasites have been reported to be immunoreactive [25], [28], [29]. EF-Tu has also been described as surface-localized, which allows it to mediate binding of *M. pneumoniae* to fibronectin [16]. In the present study, *M. bovis* EF-Ts was found to be immunoreactive. The issues of whether EF-Ts is antigenic conserved or involved in the adherence to host cells merit further study.

Pyruvate dehydrogenase (E1), dihydrolipoyl transacetylase (E2), and dihydrolipoyl dehydrogenase (E3) catalyze the conversion of pyruvic acid to acetyl CoA. With other cofactors, they form pyruvate dehydrogenase complex (PDHC). This conversion is a bond linking glycolysis and the tricarboxylic acid cycle. E1 is an α2β2 tetramer. It mainly catalyzes decarboxylation of pyruvate. Some studies have demonstrated that mycoplasma PDHB (beta subunit of E1) is a phosphoprotein with a cytoskeleton-like structure, and it can also be expressed on the surfaces of mycoplasma cells. Its primary roles are biosynthetic and metabolic and take place in the cytoplasm, but it is also involved in binding to the surface of the host cell. Previous studies have suggested that the conformation that PDHB assumes on the surface of the membrane may confer biological and virulence-related functions [16], [17]. Zhao et al. identified nine proteins, including PDHB, in the membrane protein fraction of *Mycoplasma capricolum subsp. capripneumoniae* using a MS system that reacted with convalescent sera in the immunoblots [22]. Pinto et al. identified five highly immunoreactive antigens, including the PDHB of the *M. hyopneumoniae* pathogenic strain 7448, using immunoproteomics [18].

The present study is the first to find *M. bovis* PDHB to exhibit excellent immunogenicity and repeatability. Homology analysis indicated that the amino acid sequences of PDHB of different *M. bovis* isolates were nearly identical (≥99.7%). With one exception, *M. bovis* PDHB had less than or equal to 66.2% homology with any other mycoplasma species isolated from cattle, sheep, or goats. It exhibited 98.2% homology with *M. agalactiae* PDHB. We examined the antigenicity of *M. bovis* PDHB by Western blot assay, the presence of specific straps observed in all eight *M. bovis* field isolates probed with the rabbit anti-rPDHB polyclonal antibody demonstrated that this protein is structurally and antigenically conserved within the *M. bovis* cluster. For other species of pathogens from ruminants, cross-reactivity was detected solely with *M. agalactiae* which infects sheep and goats. *M. agalactiae* is considered to be the classical agent of contagious agalactia, which occurs worldwide and is one of the principal mycoplasmoses of sheep and goats. *M. bovis* and *M. agalactiae* are closely related both phenotypically and genotypically. *M. bovis* was once called *M. agalactiae* subsp. bovis [5]. They share a 16S rDNA similarity of 99.8% and an unusually high number of related antigens and common epitopes. The existence of cross reactivity is not unexpected between *M. bovis* and *M. agalactiae.* However, epidemiological investigations have shown *M. bovis* to be generally host-specific. Although *M. agalactiae* has been isolated from cattle on extremely rare occasions, the roles of *M. bovis* and *M. agalactiae* as pathogens outside cattle and small ruminants, respectively, have yet to be defined [30]. Most studies indicated that the probability of cross-reaction between *M. bovis* and *M. agalactiae* is very low in clinical situations. Therefore, this cross-reactivity is expected to have a negligible effect on the serologic diagnosis of *M. bovis* infection in most of the countries in which farms that raise both cattle and small ruminants are rare [10].

Based on these considerations, an iELISA with rPDHB as a diagnostic antigen was established to detect anti-*M. bovis* antibody. A commercial ELISA kit was used to assess the detection rate and sensitivity of the rPDHB-based iELISA. The comparative analysis between the two methods was carried out by testing 358 serum samples, the detection rate of the iELISA (50.8%) was higher than that of the commercial kit (39.9%), even though the two methods showed a good consistency (kappa  =  0.783, where 1.0 is perfect consistency). Furthermore, rPDHB-based iELISA showed a significantly higher detection rate than the commercial kit at a serum dilution ratio of 1∶2560 to 1∶10,240, suggesting that rPDHB-based iELISA would be a better method for clinical detection with the higher sensitivity. In terms of the specificity of rPDHB-based iELISA, no cross-reaction was detected with the positive sera of other pathogens except *M. agalactiae*. As described above, the cross-reaction between *M. bovis* and *M. agalactiae* should not limit the establishment of this new ELISA detection method for *M. bovis*.

In conclusion, 19 immunogenic proteins of *M. bovis* were identified for the first time using 2-DE and MALDI-TOF/TOF MS approaches. An iELISA used to detect serum antibodies of *M. bovis* was established with rPDHB expressed in a prokaryotic system. It can provide the supplementary diagnosis to the infectious *Mycoplasma bovis* pneumonia in cattle and advance the associated epidemiological investigation and quarantine.

## Materials and Methods

### Ethics statement

All animal research was approved by the Beijing Association for Science and Technology (approval ID SYXK (Beijing) 2007-0023) and was in compliance with Beijing Laboratory Animal Welfare and Ethics guidelines as issued by the Beijing Administration Committee of Laboratory Animals. All animal studies were also performed in accordance with the China Agricultural University Institutional Animal Care and Use Committee guidelines (ID: SKLAB-B-2010-003) and approved by animal welfare committee of China Agricultural University.

### Mycoplasmas and culture conditions

*M. bovis* strain PD was used in this study. It was isolated in Shandong Province and stored in the laboratory. It was cultivated at 37°C in PPLO broth (BD, U.S.) containing 2.5% yeast extract (w/v), 20% horse serum (v/v), 0.5% phenol red (v/v) and 2000 IU/l penicillin. pH was maintained at 7.4–7.6.

### Preparation of serum samples

The 358 serum samples and the corresponding nasal swabs were collected from cattle feedlots in Beijing, Shandong, Hebei, Tianjin, Hubei, Hunan, and Heilongjiang, all of which are in China. Some of these samples were taken from cattle that showed symptoms of *M. bovis* infection, such as fever, arthritis, mastitis, conjunctivitis, and pneumonia. PCR amplification and commercial ELISA (*Mycoplasma bovis* Antibody Test Kit, BioVet, Canada) were carried out separately on all samples [31], [32]. The serum samples were kept at –70°C for further study of screening immunogenic protein and establishment of a method of detection.

### Extraction of the whole-cell proteins

A modified version of a previously described method was used to extract *M. bovis* proteins [33]. Briefly, cells were harvested at a density of 10^8^ CFU/ml by centrifugation at 4°C and 12,000 g for 20 min and then resuspended in lysis buffer (7 M urea, 2 M thiourea, 4% (w/v) CHAPS, 1% (w/v) DTT, 1% (v/v) cocktail, 0.5% (v/v) IPG buffer and 40 mM Tris-base, pH 9.6). Then cells were sonicated for 10 min on ice with a Sonifier 750 (Branson Ultrasonics Corp., U.S.). The proteins were collected by centrifugation at 4°C, 100,000 g for 1 h and lysed in the same lysis buffer at room temperature (RT) for 1 h. The protein concentration was determined using a 2-D Quant Kit (GE Healthcare, U.S.).

### 2-DE

Isoelectric focusing (IEF): IEF was performed as described previously [34]. Briefly, each pH 3–10 IPG strip (17 cm, NL) or pH 4–7 IPG strip (7 cm, NL) was rehydrated at RT for 12 h with 350 µg or 100 µg *M. bovis* protein sample in 400 µl or 150 µl rehydration sample buffer (6 M urea, 2 M thiourea, 4% (w/v) CHAPS, 65 mM DTT, 0.5% (v/v) IPG, 0.04% (w/v) Bromophenol blue and 40 mM Tris-base, pH 9.6). IEF was performed in a PROTEAN® IEF System (Bio-Rad, U.S.). The parameters used for IEF were as follows: 50 V for 2 h, 500 V for 30 min, 1000 V for 30 min, 8000 V for 4 h. The final phase of 8000 V was terminated after 50,000 Vh.

SDS-PAGE: After IEF, the IPG strips were successively equilibrated for 15 min in equilibration buffer I containing 64.8 mM DTT and buffer II containing 135 mM iodoacetamide. Two equilibrated IPG strips were subjected to 12% polyacrylamide gel electrophoresis and sealed with 0.5% agarose solution. The second dimension was carried out at 50 V for 3 h followed by 100 V for 15 h. One strip gel was stained with Coomassie brilliant blue R-350, and the other was subjected to immunoblotting. The stained gel was scanned with an Image Scanner (Amersham Biosciences, U.S.) and analyzed using the PDQuest Basic 8.0 program (Bio-Rad). Three replicates were performed.

### Immunoblotting

The separated protein spots from 2-DE gels were electroblotted onto PVDF membranes (Amersham Biosciences) using a trans-blot semi-dry transfer cell (Bio-Rad, U.S.). Blotted membranes were blocked with 10% (v/v) sheep serum (Macgene, China) in PBST (0.5% v/v Tween). Then the membranes were incubated with four anti-*M. bovis* positive sera tested with both a commercial kit and PCR (Table S1) diluted 1:800 at 4°C overnight, respectively. Three replicates were performed with each positive serum. HRP-conjugated sheep anti-bovine IgG (Sigma, USA; 1∶8000 dilution) was used as the secondary antibody at RT for 30 min. Finally, the membranes were treated with Super Enhanced Chemiluminescent Substrate (ECL) Plus (Applygen, China) in accordance with the manufacturer’s instructions. Sera from healthy cattle served as negative controls.

### MALDI-TOF/TOF MS and bioinformatic analysis

Immunoreactive protein spots were manually excised from Coomassie stained gels and in-gel digested with trypsin. Briefly, gel pieces were destained with 30% acetonitrile (ACN) in 100 mM NH_4_HCO_3_ and dried in vacuum centrifuge at RT. Then they were digested overnight in 12.5 ng/µl trypsin in 25 mM NH_4_HCO_3_ at 37°C. The dry peptide samples were reconstituted in 2 µl standard diluent (20:80 ACN:water) and spotted on a 384-well Opti-TOF stainless steel plate. They were then covered with 0.5 µl oversaturation cyano-4-hydroxy-cinnamic acid (CHCA) in 50% ACN and 0.1% trifluoroacetic acid (TFA). MS and MS/MS were performed using a MALDI-TOF/TOF instrument (4800 proteomics analyzer, Applied Biosystems). The parameters were set using the 4000 Series Explorer software (Applied Biosystems). The MS spectra were recorded in reflector mode at masses ranging from 800 to 4000 with a focal mass of 2000. MS involved a CalMix5 standard to calibrate the instrument (ABI 4700 Calibration Mixture). Combined peptide mass fingerprinting (PMF) and MS/MS queries were performed using the MASCOT search engine 2.2 (Matrix Science, Ltd., U.S.) embedded into GPS-Explorer Software 3.6 (Applied Biosystems), which had been downloaded from NCBI database (Taxonomy: Mycoplasma (7442 sequences), Dec. 10, 2010). The peptide mass tolerance was 100 ppm. MS/MS fragment tolerance was set to 0.4 Da. A GPS Explorer protein confidence index of ≥95% was used for further manual validation.

The corresponding protein sequence was downloaded from NCBI. Then the protein function classification was determined by comparison to COGs database with RPS-BLAST program, and the subcellular localization was predicted by PSORTb version 3.0 software.

### Expression of rPDHB

Sequence analysis showed *M. bovis* PDHB to contain a UGA codon. This codon is translated as tryptophan according to mycoplasma genetic code but translated as a stop codon in the *E. coli* expression system. To avoid the production of truncated gene products, this UGA codon was mutated to UGG using two PCR runs by standard overlap extension PCR (39). Two restriction enzyme cutting sites, Nco I and Xho I, were added to the flanks of the PDHB gene sequence. The mutagenic primers and flanking primers are as shown in Table 5.

**Table 5:** Primers used for expression of rPDHB in the present study.

Name	Sequence^a^	Description
MbPDHB-F	5′-TGGAAAAAATTTCATTAAATAAC	Forward primer specific to the PDHB gene of *M. bovis*
MbPDHB-R	5′-TTAGCCTAACATTTCTTCGATAAC	Reverse primer specific to the PDHB gene of *M. bovis*
MbPDHB-A	5′-CATGCCATGGCAGAAAAAATTTCATTAAATAAC	Forward primer containing a Nco I site for cloning into pET-28a (+)
MbPDHB-B	5′-CAGAAATTGGTGAGTCCCATACTCTTTGGTC	Primer for site-directed mutagenesis
MbPDHB-C	5′-GGTGACCAAAGAGTATGGGACTCACCAATTTC	Primer for site-directed mutagenesis
MbPDHB-D	5′-CGGCTCGAGGCCTAACATTTCTTCGATAAC	Reverse primer containing a Xho I site for cloning into pET-28a (+)

a The restriction endonuclease sites are underlined.

Then PDHB gene fragment was cloned into a pET-28a(+) prokaryotic expression vector to construct a pET-28a(+)-PDHB plasmid. Transetta (DE3) chemically competent cell (TransGen Biotech, China) was used to express the recombinant protein. Briefly, the competent cells carrying pET-28a(+)-PDHB plasmids were grown in Luria-Bertani (LB) media at 37°C for 3 h and induced with 1 mM isopropyl β-D-thiogalactoside (IPTG) for 4 h. Then the harvested cells were disrupted by sonication and recombinant proteins were purified by affinity chromatography to Ni-NTA columns as directed by the manufacturer (Qiagen, Germany).

### Preparation of polyclonal antibody against rPDHB

Three New Zealand white rabbits aged 6–7 weeks were injected subcutaneously with purified rPDHB (0.5 mg/kg) three times at 2-week intervals. rPDHB was blended with the same volume of complete Freund’s adjuvant (CFA, Sigma) in the first immunization and with incomplete Freund’s adjuvant (IFA) in the following immunization. A rabbit injected with adjuvants alone served as a negative control. Sera were collected two weeks after the last immunization and the antibody titers were tested using ELISA. The positive sera were either tested for specificity by Western blot analysis or stored at –70°C.

### Establishment of rPDHB-based iELISA

Indirect ELISA was performed as described previously [10]. Briefly, 96-well ELISA plates were coated with 25, 50, 100, 200, or 400 ng/well rPDHB and allowed to incubate at 4°C overnight. Sheep serum, swine serum, horse serum, protein-free blocking buffer, and gelatin served as blocking buffers. Sera (primary antibodies) were diluted to 1:40, 1:80, and 1:160–1:2560 (v/v). HRP-conjugated sheep anti-bovine IgG secondary antibody was diluted to 1∶1000, 1∶2000, 1∶4000, and 1∶8000 (v/v). Finally the substrate TMB and 2 M H_2_SO_4_ was added for coloration and termination of the reaction, respectively. The plates were read at an optical density of 450 nm (OD_450_) with a reference filter of 630 nm in an ELISA plate reader (Pharmacia, U.S.).

Fifty OD_450_ values of negative sera presenting normal distribution were used to calculate the mean optical density (OD) and standard deviation (SD). The cutoff value between positive and negative sera was calculated as the mean OD of the fifty negative sera plus 3 SDs of the mean. This calculation provides 99% confidence that all negative values fell within the defined range [35], [36].

### Specificity

Two methods were used to confirm the specificity of *M. bovis* PDHB: i) Whole-cell proteins from *M. agalactiae* (CVCC 344), *M. bovirhinis* (ATCC 27748), *M. ovipneumoniae* (ATCC 29419), BVDV (CVCC AV-69), BPIV3 (ATCC VR-281), IBRV (CVCC AV-346), and eight *M. bovis* strains were used to assess cross-reactivity with anti-rPDHB polyclonal antibodies in Western blot assays. The eight *M. bovis* strains were *M. bovis* PG45 (ATCC 25523), *M. bovis* Hubei-1 (donated by China Animal Health and Epidemiology Center), *M. bovis* SD-2 (donated by China Animal Health and Epidemiology Center) and five strains preserved in the lab, specifically *M. bovis* PD, *M. bovis* HRB-1, *M. bovis* GY-7, *M. bovis* GY-14, and *M. bo*vis WF-3. ii) rPDHB-based iELISA was used to detect three rabbit polyclonal antibodies against *M. bovirhinis*, *M. agalactiae*, and *M. ovipneumoniae* (prepared using the method described above), and four positive sera of *Mmm*SC, BVDV, BPIV3, and IBRV that had been purchased from Real Bio-technology (China). All protein samples were boiled before being loaded onto the SDS-PAGE gel. The parameters used to transfer the gel to the PVDF membrane included 60 V for 2 h. All the pathogens described above were identified using specific PCR. The primer sequences and the references are given in Table S2.

### rPDHB-based iELISA and commercial kit

The 358 serum samples were tested using rPDHB-based iELISA under optimized conditions, and the degree of agreement and sensitivity between the iELISA and the commercial kit were determined.

Sensitivity: 140 positive serum samples and 20 negative controls were randomly selected. Twofold serial dilutions of the sera from 1:80 to 1:20,480 were employed in the test. The commercial kit and rPDHB-based iELISA were used separately for detection. Samples with OD_450_ values greater than or equal to twice that of the negative serum were considered positive (A 630 nm filter served as a reference filter).

### Statistical analysis

The degree of agreement between the commercial kit and rPDHB-based iELISA was measured using kappa statistics [37]. The sensitivity of the detection of differences between the rPDHB-based iELISA and commercial kit were analyzed using the Chi-square or Fisher’s Exact test with SPSS v19.0 software. *P* values below 0.05 were considered statistically significant.

## Supporting Information

Figure S1 **Antigenicity analysis of** ***M. bovis*** **PDHB.** *M. bovis* rPDHB (lane 1) and the whole-cell proteins of *M. bovis* (lane 2), *M. agalactiae* (lane 3), *M. bovirhinis* (lane 4), *M. ovipneumoniae* (lane 5), BVDV (lane 6), BPIV3 (lane 7) and IBRV (lane 8) were separated by SDS-PAGE, blotted onto a PVDF membrane and subjected to the following Western blot analysis with rabbit anti-*M. bovis* rPDHB polyclonal antibody.
(TIF)

Figure S2 **PCR identification of the pathogens used in the present study.** Photograph of a 1% agarose gel loaded with the PCR or RT-PCR (for RNA viruses: BVDV and BPIV3) products. M: molecular weight marker. Lane 1-7: *M. bovis*, *M. agalactiae*, *M. bovirhinis*, *M. ovipneumoniae*, BVDV, BPIV3, and IBRV, respectively. The specific primers are listed in Table S2.
(TIF)

Table S1 **Positive bovine sera used in the immunoblot assays.**
(DOC)

Table S2 **Primer sequences used for PCR identification.**
(DOC)

## References

[1] Hale HH, Helmboldt CF, Plastridge WN, Stula EF (1962) Bovine mastitis caused by a Mycoplasma species. Cornell Vet 52: 582–591. 13952069

[2] Pfutzner H, Sachse K (1996) *Mycoplasma bovis* as an agent of mastitis, pneumonia, arthritis and genital disorders in cattle. Rev Sci Tech 15: 1477–1494. 9190022 10.20506/rst.15.4.987

[3] Xin JQ, Li Y, Guo D, Song NH, Hu SP, et al (2008) First isolation of *Mycoplasma bovis* from calf lung with pneumoniae in China. Chinese Journal of Preventive 30: 661–664.

[4] Tschopp R, Bonnemain P, Nicolet J, Bumens A (2001) Epidemiological study of risk factors for *Mycoplasma bovis* infections in fattening calves. Schweiz Arch Tierheilkd 143: 461–467. 11593902

[5] Nicholas RA, Ayling RD (2003) *Mycoplasma bovis*: disease, diagnosis, and control. Res Vet Sci 74: 105–112. 12589733 10.1016/s0034-5288(02)00155-8

[6] Caswel JL. Arehambauh M (2007) *Mycoplasma bovis* pneumonia in cattle. Anim Health Res Rev 8: 161–186. 18218159 10.1017/S1466252307001351

[7] Lysnyansky I, Rosengarten R, Yogev D (1996) Phenotypic switching of variable surface lipoproteins in *Mycoplasma bovis* involves high-frequency chromosomal rearrangements. J Bacteriol 178: 5395–5401. 8808927 10.1128/jb.178.18.5395-5401.1996 PMC178356

[8] Nussbaum S, Lysnyansky I, Sachse K, Levisohn S, Yogev D (2002) Extended repertoire of genes encoding variable surface lipoproteins in *Mycoplasma bovis* strains. Infect Immun 70: 2220–2225. 11895991 10.1128/IAI.70.4.2220-2225.2002 PMC127842

[9] Sachse K, Pfutzner H, Heller M, Hanel I (1993) Inhibition of *Mycoplasma bovis* cytadherence by a monoclonal antibody and various carbohydrate substances. Vet Microbiol 36: 307–316. 7505986 10.1016/0378-1135(93)90097-q

[10] Robino P, Alberti A, Pittau M, Chessa B, Miciletta M, et al (2005) Genetic and antigenic characterization of the surface lipoprotein P48 of *Mycoplasma bovis* . Vet Microbiol 109: 201–209. 15985342 10.1016/j.vetmic.2005.05.007

[11] Scherm B, Gerlach GF, Runge M (2002) Analysis of heat shock protein 60 encoding genes of mycoplasmas and investigations concerning their role in immunity and infection. Vet Microbiol 89: 141–150. 12243891 10.1016/s0378-1135(02)00158-x

[12] Prysliak T, van der Merwe F, Perez-Casal J (2013) Vaccination with recombinant *Mycoplasma bovis* GAPDH results in a strong humoral immune response but does not protect feedlot cattle from an experimental challenge with *M. bovis* . Microb Pathog 12: 1–8. 10.1016/j.micpath.2012.12.001 23246808

[13] Jaffe JD, Berg HC, Church GM (2004) Proteogenomic mapping as a complementary method to perform genome annotation. Proteomics 4: 59–77. 14730672 10.1002/pmic.200300511

[14] Ueberle B, Frank R, Herrmann R (2002) The proteome of the bacterium *Mycoplasma pneumoniae*: comparing predicted open reading frames to identified gene products. Proteomics 2: 754–764. 12112859 10.1002/1615-9861(200206)2:6<754::AID-PROT754>3.0.CO;2-2

[15] Jores J, Meens J, Buettner FF, Linz B, Naessens J, et al (2009) Analysis of the immunoproteome of *Mycoplasma mycoides subsp. mycoides* small colony type reveals immunogenic homologues to other known virulence traits in related Mycoplasma species. Vet Immunol Immunopathol 131: 238–245. 19443045 10.1016/j.vetimm.2009.04.016

[16] Dallo SF, Kannan TR, Blaylock MW, Baseman JB (2002) Elongation factor Tu and E1 β subunit of pyruvate dehydrogenase complex act as fibronectin binding proteins in *Mycoplasma pneumoniae* . Mol Microbiol 46: 1041–1051. 12421310 10.1046/j.1365-2958.2002.03207.x

[17] Su HC, Hutchison CA 3rd, Giddings MC (2007) Mapping phosphoproteins in *Mycoplasma genitalium* and *Mycoplasma pneumoniae* . BMC Microbiol 7: 47–63. 17605819 10.1186/1471-2180-7-63 PMC1947986

[18] Pinto PM, Chemale G, de Castro LA, Costa AP, Kich JD, et al (2007) Proteomic survey of the pathogenic *Mycoplasma hyopneumoniae* strain 7448 and identification of novel post-translationally modified and antigenic proteins. Vet Microbiol 121: 83–93. 17182197 10.1016/j.vetmic.2006.11.018

[19] Soehnlen MK, Aydin A, Lengerich EJ, Houser BA, Fenton GD, et al (2011) Blinded, controlled field trial of two commercially available *Mycoplasma bovis* bacterin vaccines in veal calves. Vaccine 29: 5347–5354. 21664397 10.1016/j.vaccine.2011.05.092

[20] Maunsell FP, Woolums AR, Francoz D, Rosenbusch RF, Step DL, et al (2011) *Mycoplasma bovis* infections in cattle. J Vet Intern Med 25: 772–783. 21745245 10.1111/j.1939-1676.2011.0750.x

[21] Maunsell FP, Donovan GA (2009) *Mycoplasma bovis* infections in young calves. Vet Clin North Am Food Anim Pract 25: 139–177. 19174287 10.1016/j.cvfa.2008.10.011

[22] Zhao P, He Y, Chu YF, Gao PC, Zhang X, et al (2012) Identification of novel immunogenic proteins in *Mycoplasma capricolum subsp. Capripneumoniae* strain M1601. J Vet Med Sci 74: 1109–1115. 22673397 10.1292/jvms.12-0095

[23] Thomas A, Leprince P, Dizier I, Ball H, Gevaert K, et al. Identification by two-dimensional electrophoresis of a new adhesin expressed by a low-passaged strain of *Mycoplasma bovis* . Res Microbiol 156: 713–718. 15950126 10.1016/j.resmic.2005.02.008

[24] Wasinger VC, Pollack JD, Humphery-Smith I (2000) The proteome of *Mycoplasma genitalium*. Chaps-soluble component. Eur J Biochem 267: 1571–1582. 10712586 10.1046/j.1432-1327.2000.01183.x

[25] Alonso JM, Prieto M, Parra F (2002) Genetic and antigenic characterisation of elongation factor Tu from *Mycoplasma mycoides subsp. mycoides* SC. Vet Microbiol 89: 277–289. 12383637 10.1016/s0378-1135(02)00258-4

[26] Alvarez RA, Blaylock MW, Baseman JB (2003) Surface localized glyceraldehyde-3-phosphate dehydrogenase of *Mycoplasma genitalium* binds mucin. Mol Microbiol 48: 1417–1425. 12787366 10.1046/j.1365-2958.2003.03518.x

[27] Perez-Casal J, Prysliak T (2007) Detection of antibodies against the *Mycoplasma bovis* glyceraldehyde-3-phosphate dehydrogenase protein in beef cattle. Microb Pathog 43: 189–197. 17689221 10.1016/j.micpath.2007.05.009

[28] Sun XM, Ji YS, Elashrama SA, Lu ZM, Liu XY, et al (2012) Identification of antigenic proteins of Toxoplasma gondii RH strain recognized by human immunoglobulin G using immunoproteomics. J proteomics 77: 423–432. 23026549 10.1016/j.jprot.2012.09.018

[29] Yang YL, Wang L, Yin JG, Wang XL, Cheng SP, et al (2011) Immunoproteomic analysis of Brucella melitensis and identification of a new immunogenic candidate protein for the development of brucellosis subunit vaccine. Mol Immunol 49: 175–184. 21943783 10.1016/j.molimm.2011.08.009

[30] Bashiruddin JB, Frey J, Konigsson MH, Johansson KE, Hotzel H, et al (2005) Evaluation of PCR systems for the identification and differentiation of *Mycoplasma agalactiae* and *Mycoplasma bovis*: a collaborative trial. Vet J 169: 268–275. 15727920 10.1016/j.tvjl.2004.01.018

[31] Hou X, Fu P, Zhang HY, Zhang YW, Wu WX (2012) Development of loop-mediated isothermal amplification for rapid detection of *Mycoplasma bovis* . Journal of Agricultural Biotechnology 20: 218–224.

[32] Tenk M, Bálint A, Stipkovits L, Biró J, Dencso L (2006) Detection of *Mycoplasma bovis* with an improved pcr assay. Acta Vet Hung 54: 427–435. 17278715 10.1556/AVet.54.2006.4.1

[33] Regula J, Ueberle B, Boguth G, Gorg A, Schnolzer M, et al (2000) Towards a two-dimensional proteome map of *Mycoplasma pneumoniae* . Electrophoresis 21: 3765–3780. 11271496 10.1002/1522-2683(200011)21:17<3765::AID-ELPS3765>3.0.CO;2-6

[34] Görg A, Weiss W, Dunn MJ (2004) Current two-dimensional electrophoresis technology for proteomics. Proteomics 4: 3665–3685. 15543535 10.1002/pmic.200401031

[35] Webster KA, Giles M, Dawson C (1997) A competitive ELISA for the serodiagnosis of hypodermosis. Vet Parasitol 68: 155–164. 9066061 10.1016/s0304-4017(96)01062-x

[36] Tiwari S, Kumar A, Thavaselvam D, Mangalgi S, Rathod V, et al (2013) Development and comparative evaluation of a plate enzyme-linked immunosorbent assay based on recombinant outer membrane antigens Omp28 and Omp31 for diagnosis of human brucellosis. Clin Vaccine Immunol 20: 1217–1222. 23761658 10.1128/CVI.00111-13 PMC3754501

[37] Landis JR, Koch GG (1977) The measurement of observer agreement for categorical data. Biometrics 33: 159–174. 843571

