# Supplementary figures and images for: Identification of Novel Immunogenic Proteins from Mycoplasma bovis and Establishment of an Indirect ELISA Based on Recombinant E1 Beta Subunit of the Pyruvate Dehydrogenase Complex
Source: PLoS One. 2014 Feb 10;9(2):e88328. doi: 10.1371/journal.pone.0088328 (PMC3919759; doi:10.1371/journal.pone.0088328)

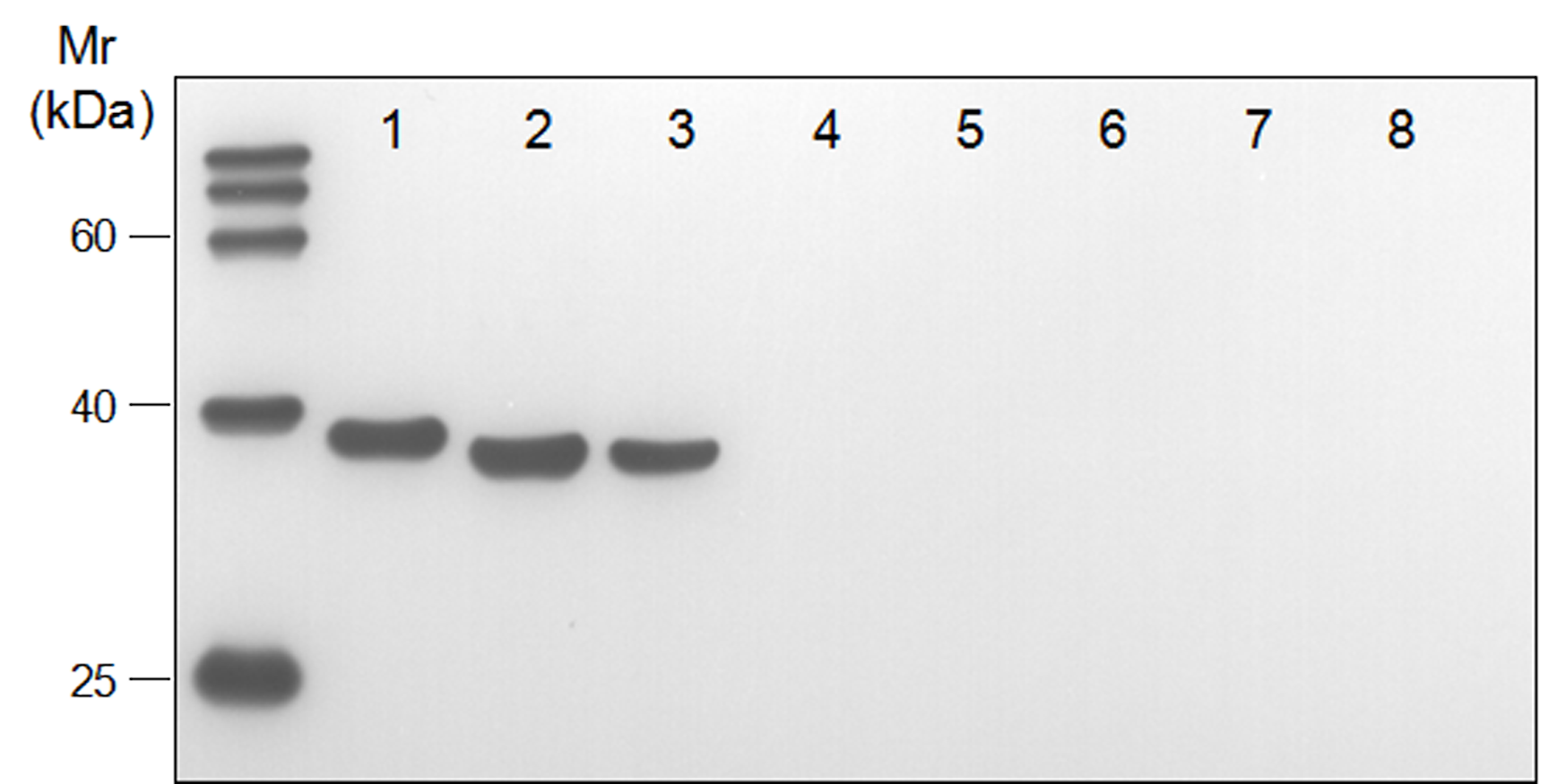

Supplement: Figure S1 — Antigenicity analysis of M. bovis PDHB. M. bovis rPDHB (lane 1) and the whole-cell proteins of M. bovis (lane 2), M. agalactiae (lane 3), M. bovirhinis (lane 4), M. ovipneumoniae (lane 5), BVDV (lane 6), BPIV3 (lane 7) and IBRV (lane 8) were separated by SDS-PAGE, blotted onto a PVDF membrane and subjected to the following Western blot analysis with rabbit anti-M. bovis rPDHB polyclonal antibody. (TIF) [file pone.0088328.s001.tif]

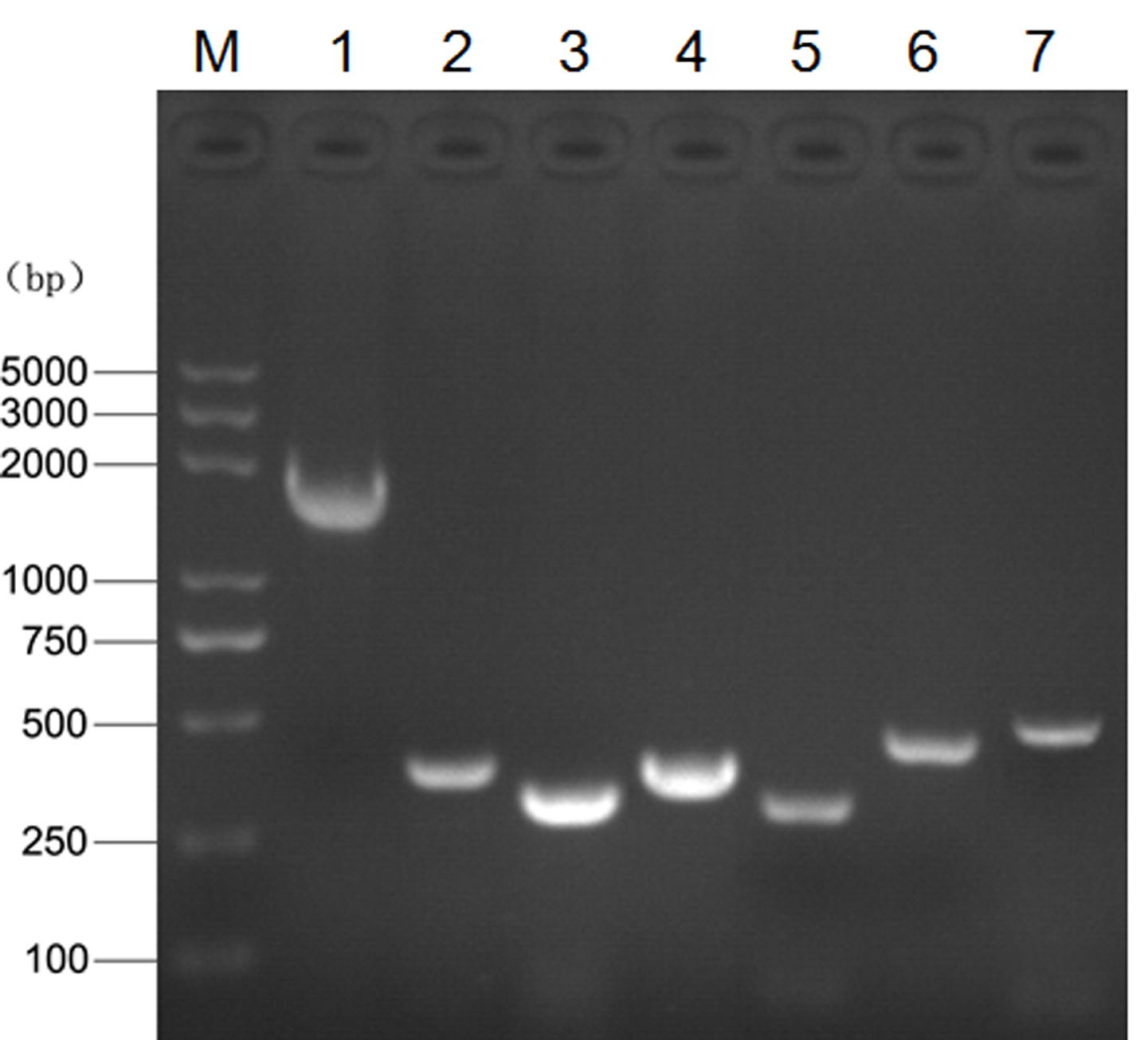

Supplement: Figure S2 — PCR identification of the pathogens used in the present study. Photograph of a 1% agarose gel loaded with the PCR or RT-PCR (for RNA viruses: BVDV and BPIV3) products. M: molecular weight marker. Lane 1-7: M. bovis, M. agalactiae, M. bovirhinis, M. ovipneumoniae, BVDV, BPIV3, and IBRV, respectively. The specific primers are listed in Table S2. (TIF) [file pone.0088328.s002.tif]
