# Supplementary material for: Identification of Novel Immunogenic Proteins from Mycoplasma bovis and Establishment of an Indirect ELISA Based on Recombinant E1 Beta Subunit of the Pyruvate Dehydrogenase Complex
Source: PLoS One. 2014 Feb 10;9(2):e88328. doi: 10.1371/journal.pone.0088328 (PMC3919759; doi:10.1371/journal.pone.0088328)
Supplement: Table S1 — Positive bovine sera used in the immunoblot assays. (DOC) [file pone.0088328.s003.doc]

**Table S1**

**Positive bovine sera used in the immunoblot assays**

| Serum sample | Origin | Year | Clinical signs in cattle | Detection results | |
| --- | --- | --- | --- | --- | --- |
|  |  |  |  | Commercial ELISA kit^a^ | PCR (nasal swabs) |
| A | Beijing, China | 2010 | Mastitis, pneumonia | 4+ | Positive |
| B | Shandong, China | 2011 | Mastitis, conjunctivitis | 4+ | Positive |
| C | Hebei, China | 2011 | Mastitis | 4+ | Positive |
| D | Heilongjiang, China | 2012 | Arthritis, pneumonia | 4+ | Positive |

^a^ Positive results were classified as 1+ to 4+ according to the kit protocol.
