# Supplementary material for: Identification of Novel Immunogenic Proteins from Mycoplasma bovis and Establishment of an Indirect ELISA Based on Recombinant E1 Beta Subunit of the Pyruvate Dehydrogenase Complex
Source: PLoS One. 2014 Feb 10;9(2):e88328. doi: 10.1371/journal.pone.0088328 (PMC3919759; doi:10.1371/journal.pone.0088328)
Supplement: Table S2 — Primer sequences used for PCR identification. (DOC) [file pone.0088328.s004.doc]

**Table S2**

**Primer sequences used for PCR identification**

| Primer | Sequence (5′-3′) | Size of specific amplicon (bp) | Specificity | Reference |
| --- | --- | --- | --- | --- |
| MbF | GGCTCTCATTAAGAATGTC | 1911 | *Mycoplasma bovis* | Hotzel et al. (1998) |
| MbR | TTTTAGCTCTTTTTGAACAAAT |
| MaF | AAAGGTGCTTGAGAAATGGC | 375 | *Mycoplasma agalactiae* | Tola et al. (1997) |
| MaR | GTTGCAGAAGAAAGTCCAATCA |
| MbrF | GCTGATAGAGAGGTCTATCG | 316 | *Mycoplasma bovirhinis* | Kobayashi et al. (1998) |
| MbrR | ATTACTCGG- GCAGTCTCC |
| MoF | TGAACGGAATATGTTAGCTT | 361 | *Mycoplasma ovipneumoniae* | McAuliffe et al. (2003) |
| MoR | GACTTCATCCTGCACTCTGT |
| BVDVF | ACGGGACAGAAGGGATACAACGGG | 294 | Bovine viral diarrhea virus | Liu et al. (2011) |
| BVDVR | CTCTTGGTGGTTGGCCCTCAGT |
| BPIV3F | GGATGTTTGGGAGTGATCTTGAGTA | 425 | Bovine parainfluenza virus type 3 | Liu et al. (2011) |
| BPIV3R | TGTGTTGAAAAATGAAGCAAGACCT |
| IBRVF | TACGACTCGTTCGCGCTCTC | 478 | Infectious bovine rhinotracheitis virus | Fuchs et al. (1999) |
| IBRVR | GGTACGTCTCCAAGCTGCCC |
